# Utilization of the halophytic shrubs *Atriplex nummularia* Lindl and *Rhagodia preissii* Moq as crops in salt-affected semi-arid regions: How temperature, salinity, seed weight and size affect seed germination

**DOI:** 10.3389/fpls.2022.989562

**Published:** 2022-10-17

**Authors:** Aslak H. C. Christiansen, Hayley C. Norman, Christian Andreasen

**Affiliations:** ^1^Department of Plant and Environmental Sciences, Faculty of Science, University of Copenhagen, Frederiksberg, Denmark; ^2^Department of Chemistry and Bioscience, Aalborg University, Aalborg, Denmark; ^3^CSIRO, Agriculture and Food, Floreat, WA, Australia

**Keywords:** dryland crops, dryland salinity, infertile land, salinity threshold, saltbush

## Abstract

The perennial halophytic shrubs *Atriplex nummularia* and *Rhagodia preissii* are native to Australia and can be planted on saline land to produce sheep and cattle feed during the autumn. However, an impediment to the adoption of the species on saline land has been the challenges in achieving successful establishment by direct seeding due to a lack of knowledge of the optimal conditions for germination. Therefore, the optimal germination requirements in relation to temperature, salinity level and seed size was assessed for each species to ascertain the optimal conditions for successful establishment by direct seeding on saline land. Seeds of both species showed optimal germination temperature at 10°C. *Atriplex nummularia* seeds were more tolerant to temperatures above or below 10°C than *R. preissii*. The germination percentage of *A. nummularia* was unchanged at 0–200 mM NaCl_2_. The germination percentage of *Rhagodia preissii* declined when the NaCl_2_ content exceeded 50 mM. There was no correlation between seed size, germination and emergence for any of the species. Based on the study, we suggest that sowing operations are performed during the cold winter months in subtropical areas or autumn and spring in temperate areas, to improve the successful establishment of these shrubs by direct seeding.

## Introduction

Increasing salinization of agricultural soils constitutes an accelerated problem. Roughly estimated 7% of the world’s agricultural soils are now salt-affected, primarily in the arid and semi-arid regions of the world making them less capable of supporting agricultural production (Wicke et al., 2011; Daliakopoulos et al., 2016). Asia, North Africa, the eastern Mediterranean area, North and South America, and Australia are most severely affected, with the highest proportion of saline lands. They are all facing a considerable loss of agricultural land due to salinity (Masters et al., 2005; Corwin, 2021). The problems with dryland salinity are particularly pronounced in the South West of West Australia (WA) where more than 1 million hectares are salt-affected and with an estimated annual loss of 14.000 hectares (Caccetta et al., 2010). The prospects for the region are further challenged by an expected increase in temperatures and extreme weather events (Furby et al., 2010; Turner et al., 2011).

Development and adoption of perennial halophytic shrubs should be promoted to increase climate resilience and maintain the profitability of otherwise unproductive saline and infertile lands (Norman et al., 2013). The additional forage provided by the shrubs can reduce the cost and reliance on supplemental grains and roughage for hand feeding during the summer/autumn feed gap, which can be a critical production cost within these livestock systems in arid and semi-arid areas (Ben Salem et al., 2010; Norman et al., 2017).

In saline regions, the cultivation of halophytic perennial shrubs has gained interest because they can produce a high-value supplemental feed for livestock on saline soils and increase water use efficacy (Glenn et al., 1999; Norman et al., 2013).

The establishment of *Atriplex nummularia, Rhagodia preissii,* and other perennial shrubs can play an important role in managing dryland salinity and counteracting erosion and degradation (Louhaichi et al., 2016). A limited number of forage crops tolerate the combined stress of salinity and arid conditions. However, species from the Chenopodiaceae family, especially *Atriplex nummularia* (Oldman saltbush), have gained attention due to their persistence in dry climates, saline, and infertile soils and their value as a supplemental feed for livestock within these areas (Le Houerou, 1992; Ben Salem et al., 2010; Norman et al., 2013). Likewise, the perennial shrub *Rhagodia preissi* (Mallee saltbush) is preferred by farmers because it performs well on nutrient-poor sandy soils, is drought tolerant and produces substantial amounts of dry matter (Kotze et al., 2011). As a result of screening potential Australian shrub species for agricultural purposes and revegetation *A. nummularia* and *R. preissii* were found to be among the shrubs with the highest biomass production (Revell et al., 2013).

A number of halophytic plants can produce high yields at elevated salinity levels. Aronson et al. (1988) studied 170 halophytes and found seven *Atriplex* species produced 12.6–20.9 t ha^−1^ of biomass containing 9.9–19.5% protein on full-strength seawater irrigation. Gallagher (1985) obtained yields of 5.2–9.5 t ha^−1^ of the saltgrass *Distichlis spicata* under seawater (30 g l^−1^) irrigation in Delaware, United States, while *Spartina patens* yielded 14.4 t ha^−1^ when harvested in July. Hollington et al. (2001) showed that saltbush (*Atriplex* spp.), bluebush (*Maireana* spp.) and other halophytic plants were also highly useful as forage in a wide range of different climatic zones of Faisalabad, Peshawar, Bhawalpur and Karachi in Pakistan. Despite the beneficial attributes of perennial halophytic shrubs in rangeland rehabilitation and livestock feeding, their adoption has been limited because of establishing costs. Commercial nursery production of seedlings was previously initiated, and seedlings were planted by tractor-drawn tree-planting machinery (Barret-Lennard et al., 1991). However, nursery-raised seedlings are expensive due to labor and nursery costs over several months, which restrict the implementation of planting (Barrett-Lennard et al., 2016). These costs are not only a barrier in South West WA but also in rangelands in West Asia and North Africa, where the establishment of these shrubs is highly sought to counter rangeland degradation (Louhaichi et al., 2019). Therefore, research in direct seeding was undertaken with the resultant development of the Mallen niche seeder (Malcolm et al., 1982), creating an optimized seedbed for germination at a much lower cost than mechanical or hand-planting of nursery-raised seedlings.

Direct seeding represents a tradeoff between cost and risk since direct seeding of fruits of *A. nummularia* only has a successful establishment rate of about 31% compared to 82% when seedlings are planted and has therefore never become a commercial reality (Barrett-Lennard et al., 2016).

The low establishment rate of direct-seeded *Atriplex* spp. is attributed to the inhibitory effects of the bracteoles surrounding the seeds. Several studies have shown that bracteole removal decreases germination time and increases the germination percentage under laboratory conditions (Stevens et al., 2006; Louhaichi et al., 2019). Therefore, removing the bracteoles is a crucial method to improve field emergence and the speed of seedling development (Ungar, 2001; Louhaichi et al., 2019). Previous studies showed that the optimal germination temperature for *A. nummularia* was 15°C (Uchiyama, 1981; Malcolm et al., 1982). It was also found that temperatures from 25°C and above reduced the germination by 50% or more and that seeds below 0.5 mg had below 10% germination. In contrast, seeds above 0.5 mg had an 83% or higher germination percentage.

No studies have examined the seed ecology of *R. preissii,* which is essential to improve direct seeding. A study of the related species *Rhagodia baccata* (berry saltbush) reported that the viability of the seeds was 63% and that the germination was highest at a temperature regime of 13/26°C compared to 18/33°C (Commander et al., 2009). Germination occurs typically at the highest water availability, and optimum germination temperature also occurs at this time of year (Bell, 1999). This indicates that optimum sowing time should be in autumn/winter, with optimal germination temperatures of 13–18°C for the Mediterranean-type species in the South West WA with winter rainfall (Bell, 1994). Seed germination in arid and semiarid regions usually occurs after the rains when the soil surface salinity has declined caused by the leaching of salts (Khan and Ungar, 1999; Li and Zhang, 2007). Several studies have shown that halophyte species have a high salt tolerance during germination but that the germination is highest in non-saline conditions and decreases with increasing salinity (Gul et al., 2013). Salinity inhibits seed germination if salinity increases above the salinity threshold of the species and causes a delay in seed germination without preventing it when the salinity level is below the species’ salinity threshold (Khan et al., 2002). Seed mass is an essential parameter for germination ability and has been found to be positively correlated with higher germination under saline conditions (Zhang et al., 2015).

Despite the efforts to achieve the successful establishment of these perennial shrubs by direct seeding to bring down the cost of establishment, there is still a lack of knowledge on the germination requirements regarding temperature, salinity tolerance and the importance of seed size. A more thorough understanding of the requirements for optimal germination has the potential to substantially improve the field emergence (Stevens et al., 2006).

This study aimed to estimate the effect of temperature and salinity on the germination of *A. nummularia* and *R. preissii* seeds. The optimal seed size for germination and seedling development was also determined to assist plant breeders in selecting appropriate material. We hypothesized that seeds of both species would have optimal germination temperatures at around 10–15°C in non-saline conditions, corresponding to the conditions in the wet winter months in Australia, where these species are naturalized and used in agriculture. We expected that seed weight was positively correlated with the total germination percentage and healthy seedling development.

## Materials and methods

### Seed collection

**Figure 1:**
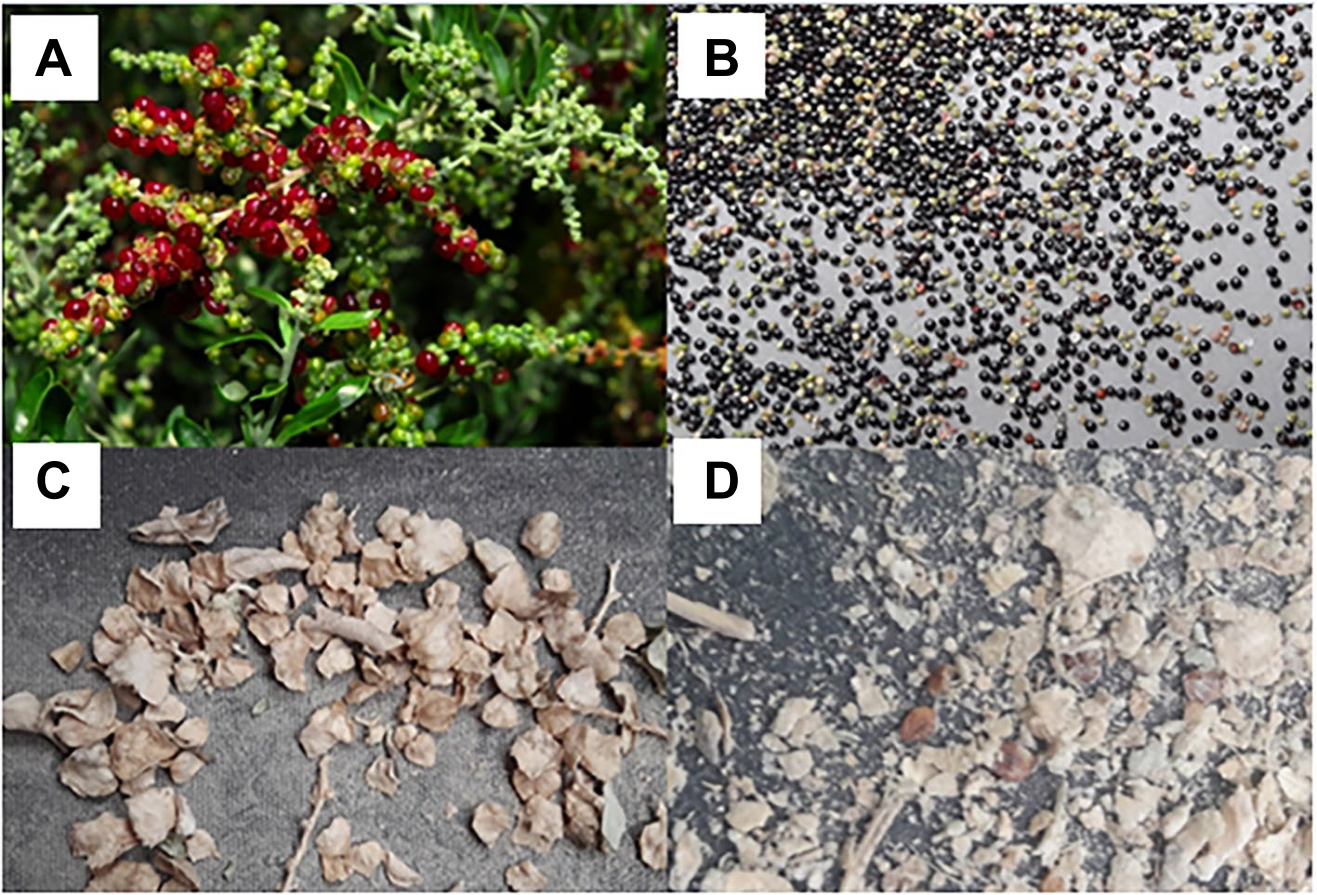
Images showing the fruits and seeds of *Rhagodia preissi* and *Atriplex nummularia*. **(A)** Developing fruits of *R. preissii* (Muirhead, 2017) that each contain one seed, **(B)** Seed of *R. preissii* both mature (black) and immature ones, **(C)** Fruits of *A. nummularia* with varying sizes of bracteoles, and **(D)** Cleaned seeds obtained by gently using corrugated rubber mats.

Seeds from a population of *R. preissii* were collected on 18 April 2019 at an experimental plot at CSIRO in Floreat, Western Australia (WA). Fruits were macerated and washed through sieves of different sizes to collect seeds. The seeds were then dried for 3 days at 40°C, and vacuum packed (Figures 1A,B).

Seeds from a naturalized population of *A. nummularia* were collected in January 2019 from a farmer’s field in Cranbrook, WA, Australia and dried for 3 days at 40°C. Pure seeds were obtained by removing the bracteoles which enclosing the seeds by gently rubbing the dried fruits between corrugated rubber matting (Figures 1C,D; Stevens et al., 2006). This is a laborious task but is necessary to obtain clean and undamaged seeds.

Four germination experiments were conducted to estimate the optimal conditions for germination of *A. nummularia* and *R. preissii* in relation to 1. temperature, 2. salinity, 3. seed size, and 4. seed weight.

For each treatment, four replicates of 100 randomly selected seeds were germinated in germination trays called the “Jacobsen apparatus” (ISTA, 2011). The apparatus consists of a transparent box with a germination plate upon which filter paper with seeds was placed. The filter paper was kept moist by a wick, extending down into an underlying water bath. Seeds were spaced uniformly and adequately apart on the filter paper. This setup ensured appropriate oxygen and water availability during the experiment (ISTA, 2011). All the germination experiments were conducted in plant growth chambers (Conviron, controlled environment systems) with 12 h of light (6 am–6 pm), and 12 h of darkness. The light source provided white light imitating natural light conditions with a photon flux density of 342 μmol m^−2^ s^−1^ of PAR (photosynthetic active radiation, 400–700 nm). Germinated seeds were counted and removed during the light period in all experiments, which lasted for a month.

### Experiment 1: Optimal germination temperature

Four replicates of 100 seeds of both species were set to germinate in plant growth chambers under four constant temperature regimes (5, 10, 20, and 30°C) to determine the optimal germination temperature. The selected temperatures are related to the range of temperatures the seeds would experience over the year in WA. Seeds were counted three times a day during the light period, and germinated seeds were removed from the filter paper. The temperature experiment was replicated once to verify the results.

### Experiment 2: Germination at different salinity levels

Seeds were placed under five different salinities of sodium chloride solution (0, 50, 100, 200, and 500 mM corresponding to a conductivity of 0, 5, 10, 20, and 50 dS m^−1^) to assess the salinity tolerance of the seeds during germination and the effect of increasing salinity on the germination percentage. Many arable soils in WA have a salt content belonging to this salinity interval. For each salinity level and species, four replicates of 100 seeds were set to germinate in plant growth chambers at 10°C. Seeds were counted three times a day during the light period. Germinated seeds were removed from the filter paper. The salinity experiment was replicated once to verify the results.

The water level in the germination trays was recorded before the experiment started and after termination. No significant water loss was recorded, indicating that there was no accumulation of salts in the germination media due to evaporation of water.

### Experiment 3: The relationship between seed weight, germination and seedling development

The correlation between individual seed weight, hours to germination and the subsequent development of seedlings were examined by weighing 300 individual seeds of *A. nummularia* and *R. preissii* on an analytical scale. The weighed seeds were placed in germination trays, and their individual position on the filter paper was noted. Germination trays were placed in a plant growth chamber at 10°C. Germinated seeds were kept in the germination tray to record the number of normal and abnormal seedlings.

### Experiment 4: The relationship between seed size and seed vigor

Seeds were sorted using metal hand seed sorters with round holes (0.75, 1, 1.25, 1.5, 1.75, and 2 mm). Seed sorters were placed on top of each other and then shaken with the seed on top for 5 min to divide them into different size fractions. Each seed sorter was thoroughly cleaned to collect all seeds. Seeds of each fraction were then counted, and the 1,000 seed weight was recorded.

Pots with holes in the bottom for drainage were filled with sand (0.4–0.9 mm), and 25 seeds were placed on the surface and covered with 5 mm sand. The pots were then placed in a plant growth chamber at 10°C. The pots were checked once a day for emerging seedlings during the light period, and pots were irrigated from below, ensuring a sufficient water supply. A seedling was characterized as fully developed when the cotyledons have fully emerged. The percentage of seeds developing a seedling was recorded.

### Statistical analyses

All experiments were analyzed separately using the open-source program R version 3.5.3.^1^ Analysis of dose–response curves was described with a three-parameter asymmetric sigmoid Weibull curve using the add-on package *drc* (Ritz et al., 2005). The seed germination data was modeled using a cumulative distribution model function of the standard log-logistic distribution (Equation 1):


(1) $$F\left(t\right)=\frac{d}{1+exp[b\{log(t)\_log(t50)\}]}$$

where *d* is the upper limit parameter that denotes the percentage of seeds that germinated during the experiment out of the initial number of seeds. *t_50_* denotes the time to germination of 50% of the maximum number of germinated seeds. The *b*-parameter is proportional to the slope of *F* at time *t* equal to the parameter *t_50._* The unit of time (hours) applied in the experiment was also applied to *t_50_*. The model checking and estimation procedures were performed by dealing with the data as event times by recording the time until germination as the event of interest (Ritz et al., 2013; Andreasen et al., 2014; Jensen et al., 2017). A significance level of 0.05 was applied in all analyses.

## Results

### Characteristics of the seeds

In general, cleaned seeds had high viability indicating that the cleaning procedure did not damage the seeds. The exact temperatures were logged to verify that the set temperature corresponded to the actual temperatures during the experimental period (Table 1).

**Table 1:** Logged temperatures during the experimental period for each of the climate chamber recorded every 5 min’s and averages presented (Standard errors = SE).

	Experiment 1		Experiment 2	
	Temperature	SE	Temperature	SE
5°C	4.7	0.1	4.7	0.2
10°C	10.7	0.2	9.5	0.1
20°C	19.4	1.1	21.0	0.4
30°C	28.5	0.2	29.6	0.4

**Figure 2:**
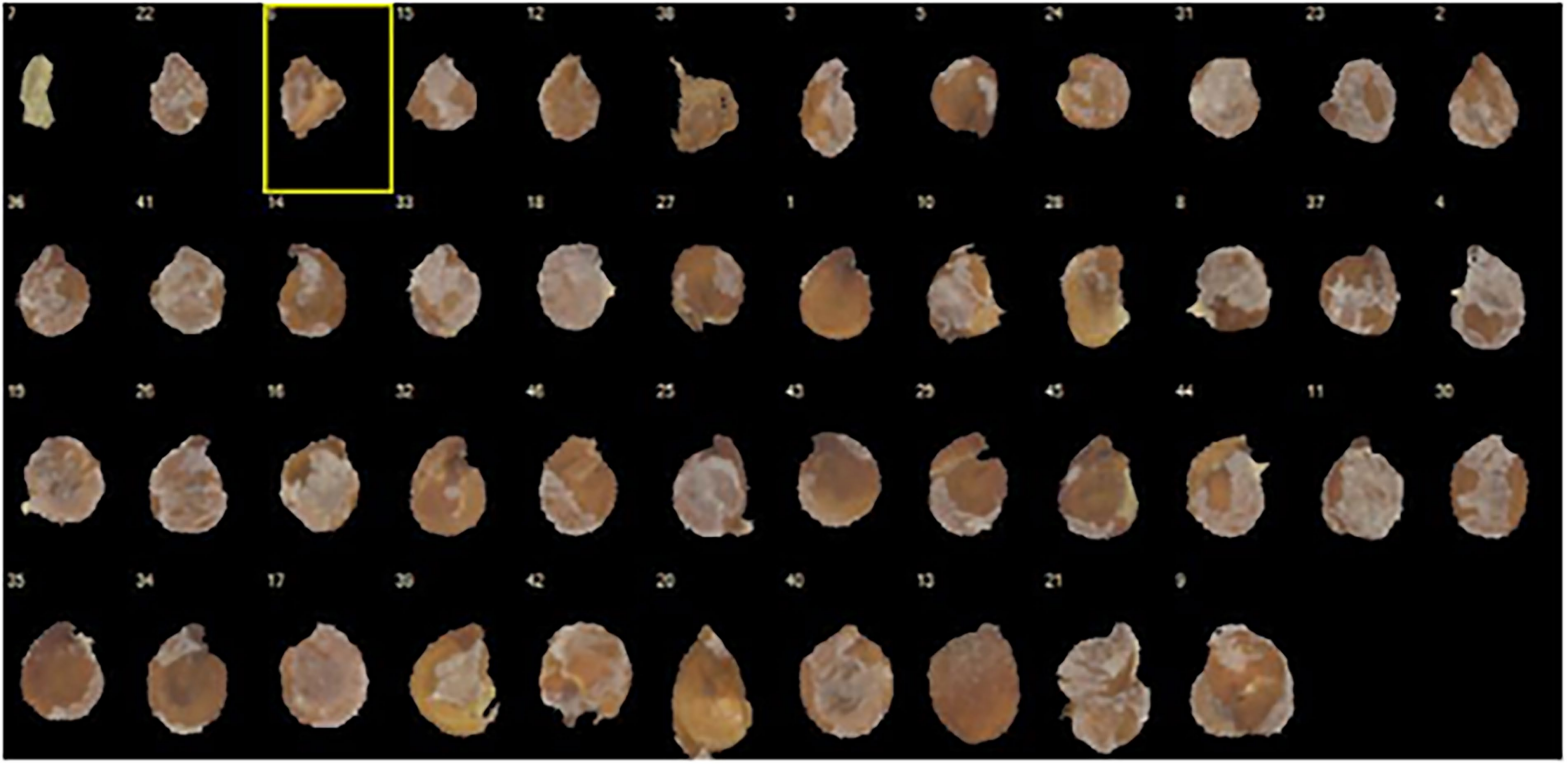
Image illustrating the large variance in seed size for *A. nummularia.*

The average 1,000 seed weight was 600 mg (SE = 53 mg) for *R. preissii* and 980 mg (SE = 41 mg) for *A. nummularia.* The variance in seed weight and size was large for seeds of *A. nummularia* (Figure 2), while the seeds of *R. preissii* were more uniform in seed size and weight (Figure 1).

### Experiment 1: Optimal germination temperature

**Figure 3:**
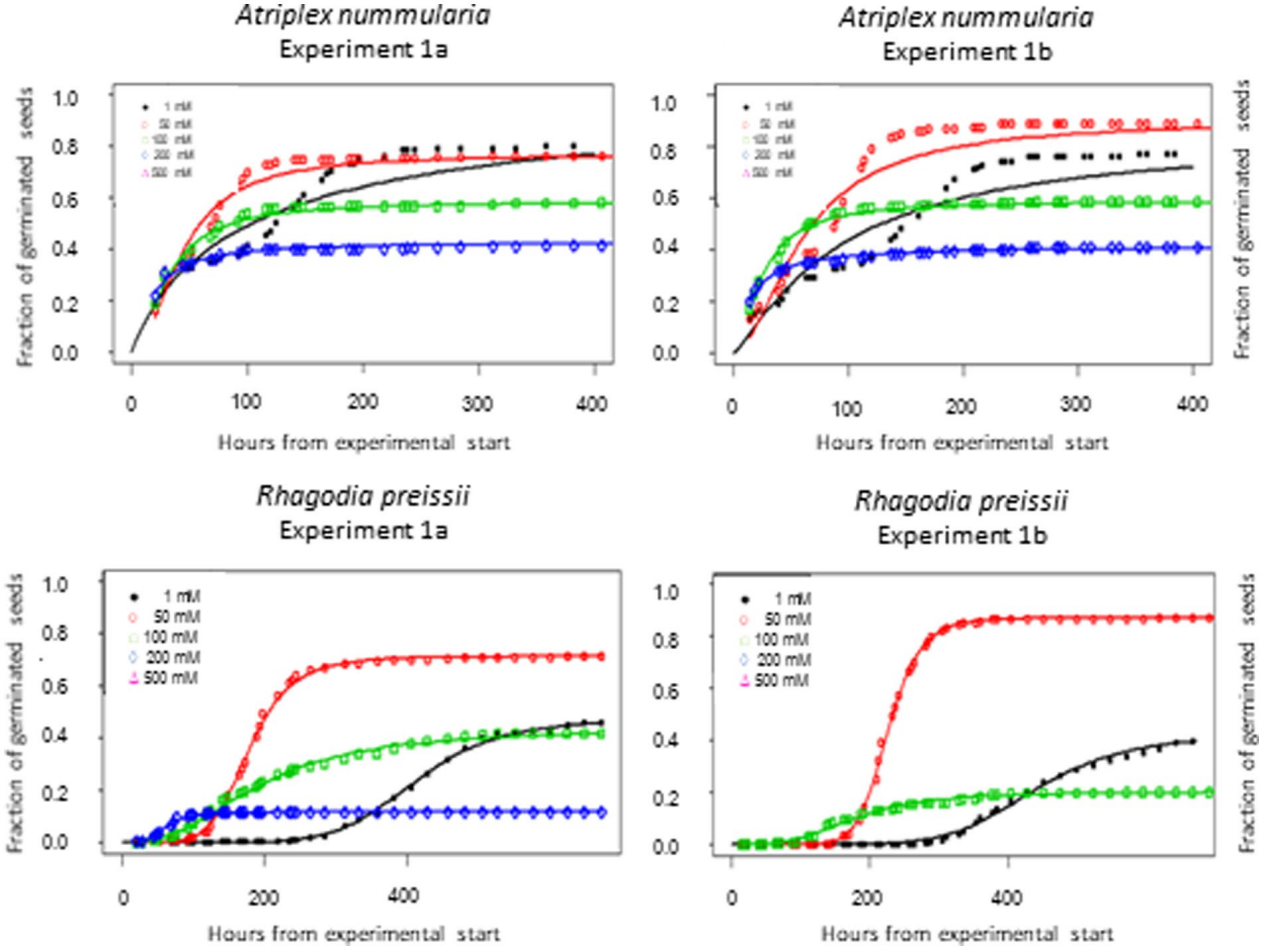
Germination curves for *A. nummularia* and *R. preissii* at four constant temperatures. In experiment 1B at 30°C no seeds of *R. preissii* germinated.

The parameter *t_50_* (time to germination of 50% of the maximum number of germinated seeds) was estimated to be the same in both temperature experiments for both species (Tables 2, 3). The maximum germination percentages were also alike (Tables 2, 3). The *b* parameter (relative slope at *t_50_*) is an expression of the speed of germination at t_50_ (Figure 3).

**Table 2:** Temperature experiment 1A: influence of temperature on seed germination of *A. nummularia* and *R. preissii* under four constant temperatures, expressed by the estimated parameters and their associated standard errors (SE) from the log-logistic model (1) applied to the data^*^.

Species	Treatment (°C)	*t_50_* (hours)		Maximum germination (%)		Relative slope	
		*t_50_*	SE	*d*	SE	–*b*	SE
*Atriplex nummularia*	5	103.1^a^*	10	0.81^a^	0.02	0.89^a^	0.05
	10	43.2^b^	2.2	0.76^b^	0.02	1.99^b^	0.11
	20	30.9^c^	2.1	0.58^c^	0.02	1.72^b^	0.12
	30	17.7^d^	2	0.42^d^	0.02	1.34^ab^	0.15
*Rhagodia preissii*	5	412.1^a^	7.8	0.47^a^	0.02	7^a^	0.51
	10	177.8^b^	3	0.71^b^	0.02	5.91^a^	0.3
	20	189.7^b^	11	0.43^a^	0.02	2.51^b^	0.19
	30	60.5^c^	3.8	0.12^c^	0.01	3.92^c^	0.51

* letters denote significant differences between the treatments.

**Table 3:** Temperature experiment 1B: influence of temperature on seed germination of *A. nummularia* and *R. preissii* under four constant temperatures, expressed by the estimated parameters and associated SE from the log-logistic model (1) applied to the data.

Species	Treatment (°C)	*t_50_* (hours)		Maximum germination (%)		Relative slope	
		*t_50_*	SE	*d*	SE	-*b*	SE
*Atriplex nummularia*	5	94.3^a,^^*^	8.5	0.84^a^	0.02	1.2^a^	0.08
	10	60.8^b^	3.2	0.90^a^	0.01	1.78^b^	0.09
	20	24.8^c^	1.7	0.68^b^	0.02	1.67^b^	0.11
	30	14.4^d^	1.8	0.41^c^	0.02	1.19^a^	0.13
*Rhagodia preissii*	5	439.3^a^	9.7	0.42^a^	0.02	6.65^a^	0.5
	10	226.1^b^	2.2	0.86^b^	0.01	9.71^b^	0.43
	20	177.9^c^	10.4	0.20^c^	0.02	3.41^c^	0.34
	30	n/a^**^	n/a	n/a	n/a	n/a	n/a

* letters denote significant differences between the treatments.

** n/a = data not available due to no germination.

The highest germination percentage occurred at 10°C for both species, with 10°C being significantly higher than the three other temperatures for *R. preissii*. The germination at 10°C was only significantly higher than at 5°C for *A. nummularia* in one of the tests, but significantly different from results at 20 and 30°C in both experiments. There was a significant decrease in *t_50_* values for both species at increasing temperatures.

### Experiment 2: Germination at different salinity levels

*t_50_* Values and maximum germination percentage were approximately the same in the Experiments 2A and 2B for both species (Tables 4, 5). The highest germination percentage for *A. nummularia* was reached at 0–200 mM, with no significant differences in Experiment 2A but some significant differences in Experiment 2B for 0–200 mM. 500 mM reduced the maximum germination significantly in both tests.

**Table 4:** Salinity experiment 2B: influence of salinity level on seed germination of *A. nummularia* and *R. preissii* at five different salinity levels, expressed by the estimated parameters and associated standard error (SE) from the log-logistic model (1) applied to the data.

Species	Treatment (mM)	*t_50_* (hours)		Maximum germination %		Relative slope	
		*t_50_*	SE	*D*	SE	*–b*	SE
*Atriplex nummularia*	0	64.5^a,^^*^	2.8	0.83^a^	0.01	2.22^a^	0.11
	50	81.5^b^	3.2	0.84^a^	0.01	2.42^a^	0.12
	100	91^c^	3.3	0.85^a^	0.01	2.56^a^	0.12
	200	114.8^d^	4.6	0.81^a^	0.02	2.43^a^	0.12
	500	143.6^e^	7.9	0.23^b^	0.01	2.94^b^	0.25
*Rhagodia preissii*	0	226.7^a^	2.6	0.74^a^	0.02	8.4^a^	0.42
	50	280.2^b^	3.9	0.75^a^	0.02	7.12^b^	0.34
	100	371.6^c^	6.2	0.65^b^	0.02	6.54^b^	0.36
	200	563.1^d^	13.2	0.49^c^	0.03	6.77^b^	0.55
	500	n/a^**^	n/a	n/a	n/a	n/a	n/a

* letters denote significant differences between the treatments.

** n/a = data not available due to no germination.

**Table 5:** Salinity experiment 2A: influence of salinity level on seed germination of *A. nummularia* and *R. preissii* at five different salinity levels, expressed by the estimated parameters and associated standard errors (SE) from the log-logistic model (1) applied to the data.

Species	Treatment (mM)	*t_50_* (hours)		Maximum germination (%)		Relative slope	
		*t_50_*	SE	*D*	SE	*–b*	SE
*Atriplex nummularia*	0	53.4^a,^^*^	2.7	0.83^a^	0.01	1.92^a^	0.09
	50	63.4^b^	3	0.81^ab^	0.02	2.07^a^	0.11
	100	73.1^c^	3.4	0.78^b^	0.02	2.13^a^	0.11
	200	140.8^d^	9.7	0.82^a^	0.02	1.24^a^	0.06
	500	139.8^e^	11.4	0.25^c^	0.02	2.13^a^	0.23
*Rhagodia preissii*	0	227.4^a^	3.7	0.64^a^	0.02	6.54^a^	0.34
	50	264^b^	3.7	0.68^ab^	0.02	7.49^a^	0.39
	100	340.3^c^	6	0.59^ac^	0.02	6.46^a^	0.36
	200	538.6^d^	12.3	0.29^d^	0.02	7.77^a^	0.71
	500	n/a^**^	n/a	n/a	n/a	n/a	n/a

* letters denote significant differences between the treatments.

** n/a = data not available due to no germination.

**Figure 4:**
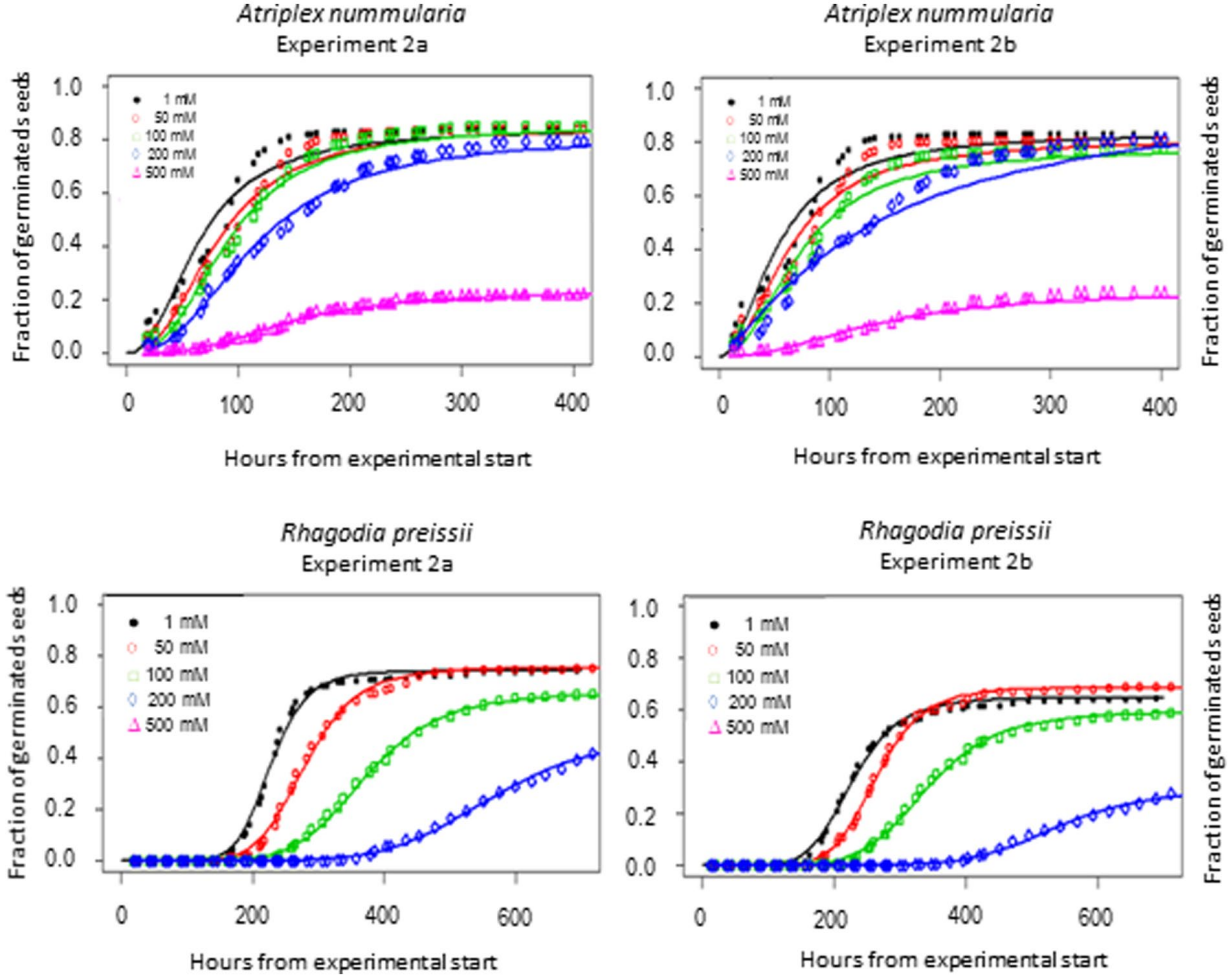
Germination curves for *A. nummularia* and *R. preissii* at five different salinity levels. No seeds of *R. preissii* germinated at the 500 mM.

*Rhagodia preissii* had maximum germination at 0–50 mM. A significant reduction occurred already at 100 mM, and was reduced even more at 200 mM (Figure 4). No *R. preissii* seeds were able to germinate at 500 mM. There was a significant increase in *t_50_* values with increasing salinity levels for both species.

### Experiment 3: The relationship between seed weight, germination and seedling development

The correlation between seed weight, germination ability and the consequent development of healthy seedlings is essential for sowing operations. The germination percentage increased with increasing seed weight above a minimum weight. *Atriplex nummularia* seeds above 0.75 mg and *R. preissii* seeds above 0.5 mg had the highest germination percentage (Table 6). For both species, there were no significant differences or trends in hours to germination with increasing seed weight. While seeds of *A. nummularia* readily germinated, seeds of *R. preissii* started germinating 6 days after.

**Table 6:** Germination percentage of individually weighed seeds sorted into weight groups, and the groups average time (hours) to germination and seedling development with standard errors (SE).

Species	Weight groups (mg)	Average seed weight (mg)	Number of seeds	Germination (%)	Hours to germination	Seedling development (%)
*Atriplex nummularia*	0–0.5	0.36 (0.005)	69	10.1	105 (3)	100
	0.5–0.75	0.65 (0.004)	33	69.7	65 (2)	83
	0.75–1	0.88 (0.004)	52	96.2	84 (3)	80
	1–1.25	1.12 (0004)	81	95.1	70 (2)	87
	1.25–1.5	1.37 (0.004)	43	97.7	63 (3)	88
	1.5–1.75	1.61(0.005)	16	100	82 (3)	94
	1.75–2	1.93(0.012)	6	100	65 (2)	83
*Rhagodia preissii*	0–0.5	0.38 (0.005)	41	7.3	232 (3)	100
	0.5–0.625	0.57 (0.002)	57	91.2	232 (3)	100
	0.625–0.75	0.69 (0.002)	103	98	227 (2)	98
	0.75–0.825	0.78 (0.001)	60	95	230 (2)	100
	0.825–1	0.89 (0.003)	39	94.9	252 (2)	92

**Figure 5:**
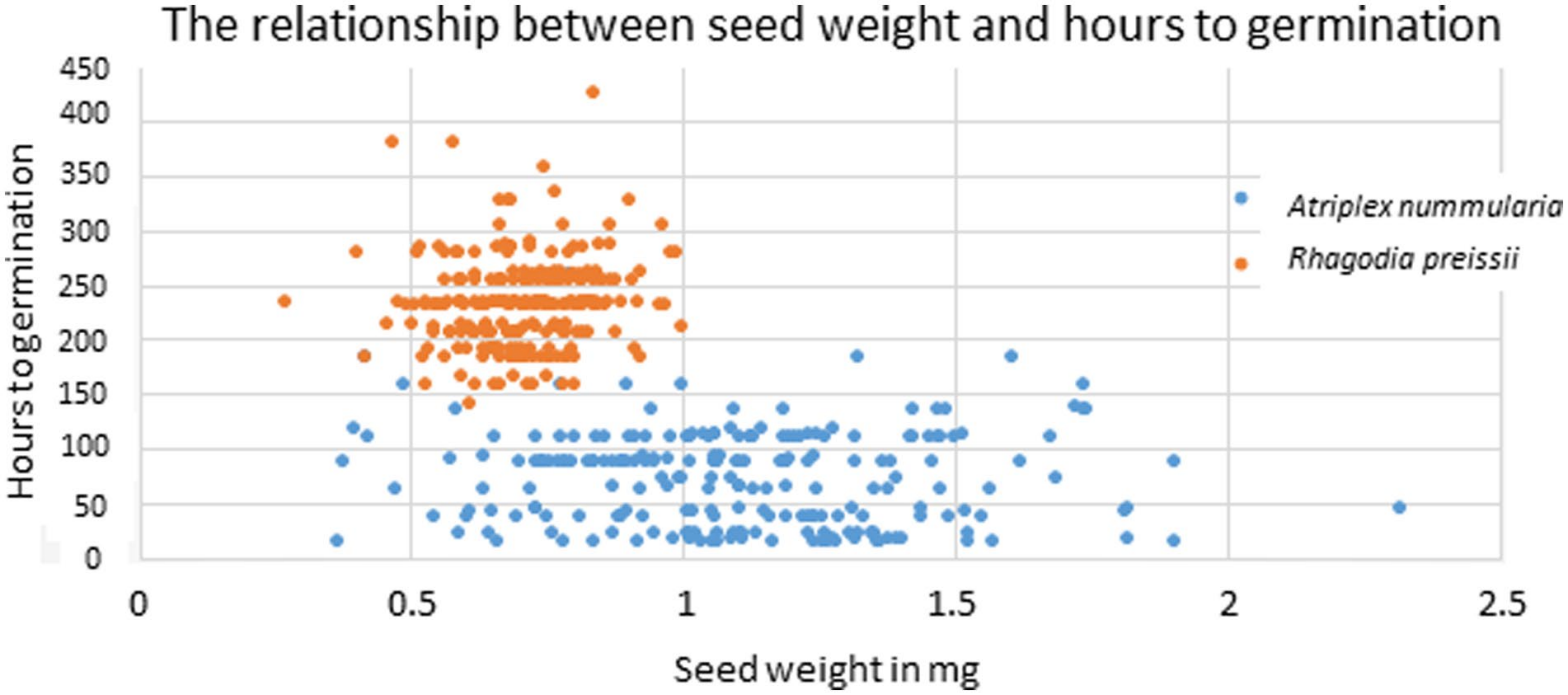
Scatter plot showing the correlation between individual seed weight and the number of hours to germination for *A. nummularia* (blue) and *R. preissii* (orange).

The uniformity in hours to germination for different seed weights was primarily caused by a high variation within each weight group, as visualized in Figure 5. On average, the variation in hours to germination within all size groups was 45 h for *A. nummularia* and 42 h for *R. preissii.*

### Experiment 4: The relationship between seed size and seed vigor

All seeds of *A. nummularia* passed through the 2 mm hand seed sorter, and roughly 90% of the seeds were between 1.25 and 1.75 mm (Table 7). All *R. preissii* seeds passed through the 2 mm, 1.75 mm, and 1.5 mm hand seed sorters. Approximately 75% of the seeds were between 0.75 and 1.25 mm. There was a correlation between seed size and 1,000 seed weight for *A. nummularia*, while this correlation was not significant for *R. preissii,* which had a more uniform seed weight and shape.

**Table 7:** The distribution of 3,000 seeds of each species into different size groups.

	*Atriplex nummularia*		*Rhagodia preissii*	
Seed size (mm)	Number of seeds	100 seed weight (mg)	Number of seeds	100 seed weight
< 0.75	159	54.9	107	58.5
< 1	49	n/a	1740	62.6
< 1.25	1,281	78.8	443	56.2
< 1.5	1,393	114.5	710	74.7
< 1.75	118	126.2	0	n/a*
< 2	0	n/a	0	n/a

* n/a = data not available due to no germination.

Seed weight values only shown for fractions with more than 100 seeds.

Seeds of *A. nummularia* emerged faster than *R. preissii* seeds, and consequently, the seedlings of *A. nummularia* were considerably larger at the end of the experiment than the *R. preissii* seedlings. There was a significant increase in *t_50_* values with increasing seed size up to 1.5 mm for *A. nummularia*, although no correlation was found between increasing size and maximum emergence. However, only seeds < 1.5 mm and seeds < 1.75 mm performed significantly differently (Table 8).

**Table 8:** The influence of seed size on the germination of *A. nummularia* and *R. preissii* expressed by the estimated parameters and their associated standard errors (SE) for each size fraction.

Species	Size fraction (mm)	*t_50_* (hours)		Maximum emergence (%)		Relative slope	
		*t_50_*	SE	*d*	SE	–*b*	SE
*Atriplex nummularia*	< 0.75	297.9^a^*	8.2	0.40^a^	0.05	9.97^a^	1.34
	< 1.25	329.8^b^	12.7	0.40^a^	0.05	7.13^ab^	0.97
	< 1.5	369.4^c^	15.4	0.51^ab^	0.05	5.94^b^	0.77
	< 1.75	345.3^bc^	16.5	0.31^ac^	0.05	6.59^b^	1.04
*Rhagodia preissii*	> 0.75	552.7^a^	13.7	0.62^a^	0.05	9.51^a^	1.16
	< 0.75	572^a^	14.1	0.70^a^	0.05	9.19^a^	1.11
	< 1	558.5^a^	14.4	0.67^a^	0.05	8.94^a^	1.09
	< 1.25	575.8^a^	17.3	0.64^a^	0.05	8.31^a^	1.12

* letters denote significant differences between the treatments.

**Figure 6:**
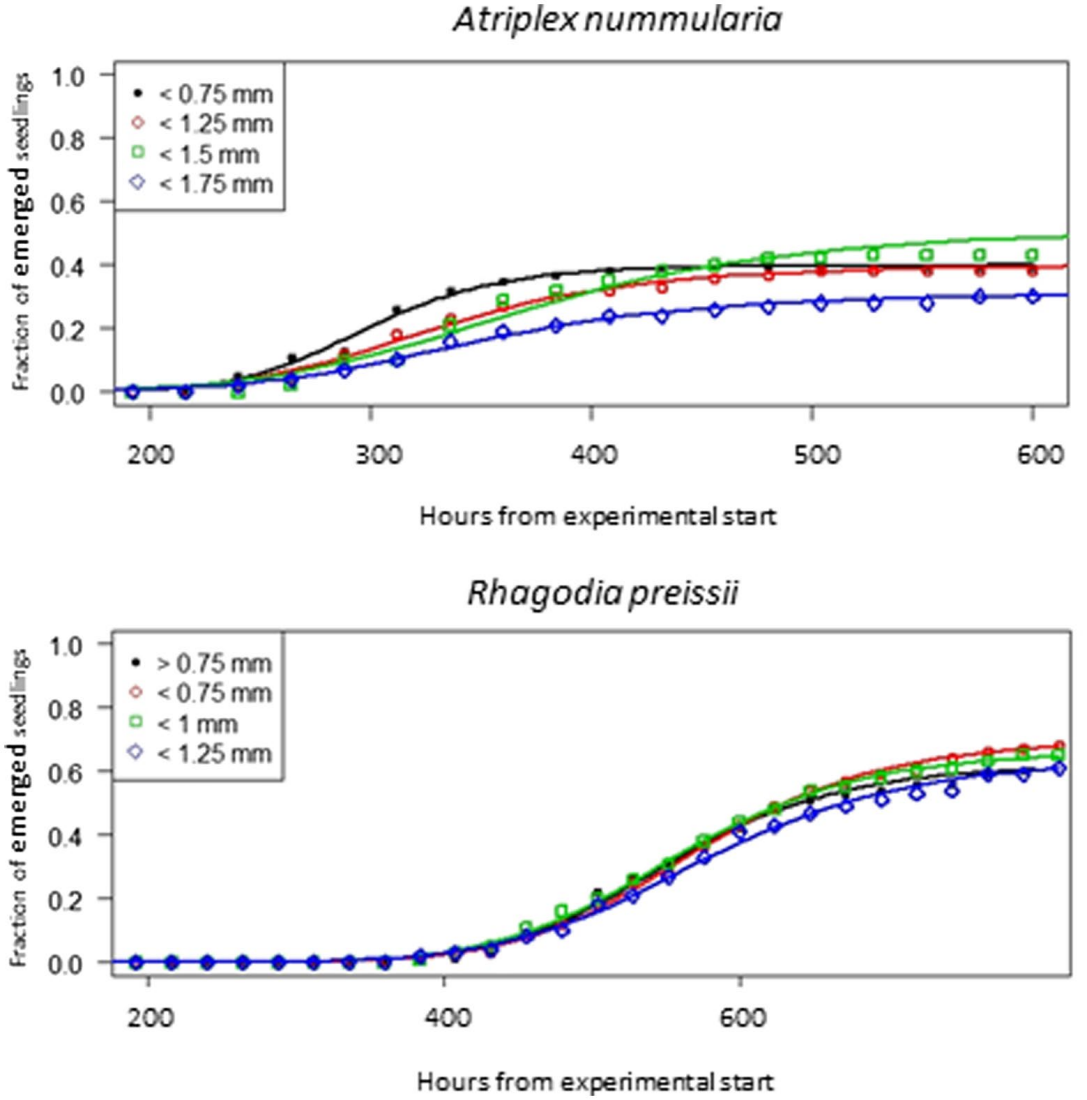
Emergence curves for four different seed size fractions of *A. nummularia* and *R. preissii* sown in 5 mm depth in sand.

The seeds of *R. preissii* had a slower emergence rate and smaller seedlings and were more uniform with no significant differences in *t_50_* values or total emergence percentage (Figure 6). The total emergence percentage was on average for all four size fractions 65.8% for *R. preissii* and 40.5% for *A. nummularia*.

## Discussion

### Temperature requirement

Generally, *A. nummularia* germinated faster than *R. preissii*, with an average *t_50_* of 52 h (2.1 days) at 10°C compared with a *t_50_* value of 202 h (8.4 days) for *R. preissii*. There was a significant decrease in *t_50_* values with increasing temperatures, indicating that the seeds germinate faster at higher temperatures. Fast germination and seedling development are required to utilize the available moisture in the soil after a rainfall event, ensuring root development and persistence through potential dry periods after germination. Although rapid germination is desired, higher temperatures come at the cost of reduced germination percentage above 10°C. *Atriplex nummularia* had similar maximum germination at 5 and 10°C (82.5 and 83%), and only 63% germination at 20°C and 41.5% at 30°C.

*Rhagodia preissii* was more sensitive to high temperatures. None or a few seeds germinated at 30°C, and only roughly 40% of the seeds germinated at 5 and 20°C compared with, on average, 78.5% germination at 10°C. The experiments were conducted at constant temperatures. However, the temperature usually fluctuates day and night, and testing different thermo-periods would probably improve the estimation of the optimal sowing time. However, it is degree days that determine the germination speed.

The tradeoff between maximum germination and germination time should be considered in relation to the risk of water stress and drying of the topsoil during germination. The tradeoff is less critical for *A. nummularia* than *R. preissii*, as *R. preissii* is more sensitive to temperatures above or below 10°C. The water availability is higher, and the temperature lower during winter, creating ideal conditions for germination. The 10-degree temperature optimum corresponds to temperatures in June to August in WA, the months with the most rainfall, indicating that the two species have naturally adapted to germinate during the winter months when the temperature is low, and the water availability is higher. In the spring, the temperatures are higher, and periods between rainfall events are greater, increasing the risk of drying out of germinating seeds before seedlings have been established.

One factor that could impair the sowing of seeds in winter at the onset of the rains could be a high soil salinity level, inhibiting and delaying germination and thereby making it more favorable to delay sowing until more salt has leached. Direct seeding is only recommended for sandy soils due to the faster leaching of salts from these soils after the onset of rains in May.

### Salinity tolerance

Experiments 2A and 2B showed that *A. nummularia* had a very high salinity threshold with no reductions in maximum germination at 200 mM, but *t_50_* increased significantly, illustrating the delay in seed germination also observed for many other species (Gul et al., 2013). At 500 mM, about 27% of *A. nummularia* seeds still germinated, a significantly higher percentage than the 10% previously reported (Uchiyama, 1987). The seeds used in this study were collected from a saline field, which may explain the difference. It has been documented that *A. nummularia* seeds collected from plants growing in a high salinity medium had a higher salt tolerance at the germination stage than seeds originating from plants grown in a low salinity medium (Uchiyama, 1987). Therefore, seeds should be collected from plants growing on saline soils for plant production on saline soils.

*Rhagodia preissii* seeds had a lower salinity threshold than *A. nummularia* seeds, with significant reductions in maximum germination starting to occur at 100 mM and significant for both salinity tests at 200 mM. The delay in germination was also higher for *R. preissii*, increasing the *t_50_* by 324 h (14 days) on average when the salinity increased from 0 to 200 mM, compared with 69 h (3 days) for *A. nummularia*. The *t_50_* increased by 242% for *R. preissii* and 221% for *A. nummularia.* Hence, the effect on *t_50_* is similar. Still, in practice, the longer delay and reduction in maximum germination can make it more difficult to successfully establish *R. preissii,* especially in soils with high salinity levels, as a concentration of 500 mM ultimately hampered the germination. Seeds of *R. preissii* were collected from a non-saline area. Therefore, it should be studied whether seeds from plants growing on more saline soils tolerate a higher salt concentration during germination.

For many halophyte species, seeds inhibited by a salinity level above the species threshold, will resume germination when the salinity level is lowered (Khan and Gul, 2006). This mechanism ensures that the seeds persist in the seed bank despite high salinity stress under high evaporative conditions. This mechanism may also count for *R. preissii*. It has been shown that *A. nummularia* seeds resume germination when the salinity level is lowered (Uchiyama, 1987). If seeds are sowed too early in the winter when the salinity is high, they will eventually start germinating when the level falls below the threshold for seed germination. However, it remains unknown how large a fraction of the seeds remain viable after enduring extreme salinities in the soil solution. The low salinity threshold and sensitivity to warmer temperatures above 10°C make the window for the sowing of *R. preissii* narrower than for *A. nummularia.*

### The relationship between seed weight, germination and seedling development

The percentage of healthy developed seedlings was highest for *R. preissii* seeds. On average, of all the germinated seeds, 98 ± 3.5% of the *R. preissii* seeds developed into a healthy seedling while 87.8 ± 7% of *A. nummularia* seeds did. Only a few germinated seeds did not develop seedlings and died after germination or developed abnormally.

For both species, there was no correlation between seed weight and the number of hours to germination. There was a considerable variation in time to germination within each weight group*. Atriplex nummularia* is an outcrossing species producing genetically different seeds, which may explain the large variation in germination time. The reproductive physiology of *R. preissii* remains unknown.

The seed weight of cleaned seeds was not essential to achieving optimal germination and seedling development. Our results indicate that if seed weight is above 0.75 mg for *A. nummularia* and 0.5 mg for *R. preissii,* most seeds are viable and able to develop into healthy seedlings, given that optimal conditions are present. Therefore, there is no reason to collect and sort only the heaviest seeds but avoid the lightest.

### The relationship between seed size on seed vigor

Despite the high percentage of healthy developed seedlings, the vigor test showed a low emergence percentage of *A. nummularia* seeds (40.5%) in the sand at a constant water level of 10°C. Stevens et al. (2006) found that the emergence percentage was considerably higher when seeds were sown at 8 mm depth in a temperature regime during the day and night of 19.4–4.4°C compared with only 5% emergence in field trials (Stevens et al., 2006). However, their field experiment was performed in September, when the temperatures are less optimal with fewer rainfalls than in the winter months.

Despite having similar germination at 10°C the emergence percentage was significantly higher for *R. preissii* (65.8% of seedlings emerged). At 10°C, there were approximately 200 h (8 days) from the first seed germinated until the first seedling emerges. The time to emergence was roughly the same for both species and each seed size fraction.

An explanation why clean seeds do not establish seedlings when they are sown in the field could be that the tiny seeds start germinating when rainfall occurs but dry out before the next rainfall. The small seed size and the consequent requirement for a low seeding depth makes the seeds prone to water stress in the early developmental stages. Our results show that despite providing optimal temperature and water availability for germination of *A. nummularia* the emerged seedling only represents 50% of the viable seeds, which germinated at 10°C in the controlled environment. The seeding depth might be responsible for the reduction in emergence percentage of *A. nummularia*. Light only increases *A. nummularia* germination by approximately 12%. A related species, *A. amnicola,* was not negatively affected by increasing the sowing depth from 5 to 10 mm (Stevens et al., 2006).

### Recommendations for direct seeding based on germination requirements

Farmers should initiate sowing operations in autumn to improve the success of direct seeding by utilizing the winter rains ensuring root development and establishment before the next dry period occurs. Sowing in the autumn should occur after the first rains have washed out most salts from the topsoil. For species growing in arid and semi-arid regions, germination usually occurs after the rain periods when the surface soil salinity has declined by the leaching of salts (Khan and Ungar, 1999). Especially *A. nummularia* can be sown successfully in soils with relatively high soil salinity (up 200 mM NaCl) without reducing the germination. *Rhagodia preissii,* on the other hand, has a narrower window for optimal germination due to lower salinity tolerance and low tolerance of temperatures above 10°C. Hence, it might be challenging to successfully establish *R. preissii* by direct seeding on clay soils because the salinity level of the soil solution may not be low enough before temperatures are too high for optimal germination.

However, earlier sowing also exposed the seeds and young plants to colder temperatures and occasional night frost during winter, compared to sowing in late winter or the spring. Both species are known to be frost tolerant, but the growth and survival rate of seedlings grown from sown seeds compared with 4 months old nursery seedlings remains to be clarified. Seeds adapted to germinate at a temperature optimum of 10°C, which occurs during winter, should also be able to persist and grow during this season. Experiments with seedlings of *A. nummularia* have indeed shown that seedling growth was less affected by high salinity levels at cold temperatures (Uchiyama, 1987).

Sowing during the autumn may be constrained by competing priorities like the sowing of annual crops. If the successful establishment of these shrubs through direct seeding are to be achieved, the importance of the timing of sowing operations needs to be communicated to farmers, extension staff and researchers intending to establish these shrubs.

## Conclusion

The seeds of both species had high viability of roughly 80% when optimal conditions were present. Seeds of *A. nummularia* and *R. preissii* had optimal germination temperatures at 10°C under non-saline conditions, confirming the proposed hypothesis. Seeds of *A. nummularia* were found to have a high salinity threshold only with significant reductions in germination at 500 mM NaCl. *Rhagodia preissii* germination was more sensitive to temperatures above or below 10°C and at salinities above 50 mM than *A. nummularia*. Experiments combining temperature and salinity levels are needed to estimate the combined effect of the two factors.

In contrast to expectations, no correlation was found between seed weight and total germination and healthy seedling development for both species when the seed weight was above 0.75 mg for *A. nummularia* and 0.5 mg for *R. preissii*. Likewise, there was no correlation between seed size and emergence for seeds sown in the sand at a depth of 5 mm. Instead of selecting only the largest and heaviest seeds, the smallest and lightest should be avoided for sowing. The emergence percentage of *R. preissii* corresponded to the number of viable seeds, but only 50% of the viable seeds of *A. nummularia* emerged due to unknown factors. Based on the required temperatures for optimal germination and the salinity tolerance of the seeds of the two species, sowing operations are recommended to be performed early during the winter months of June and July in WA. Sowing of *R. preissii* would be recommended to be performed on soils with less salinity or later in the winter.

## Data availability statement

The original contributions presented in the study are included in the article/supplementary material, further inquiries can be directed to AC, aslakheuser@gmail.com.

## Author contributions

AC did the experimental work and the statistics and wrote the first draft for the article. HN and CA supervised the work during the project and improved the manuscripts. All authors contributed to the article and approved the submitted version.

## Funding

The research was partially funded by University of Copenhagen and by scholarships from William Demant Foundation, Knud Højgaards Foundation, and Ingeniør Svend G. Fiedler og Hustrus foundation to Promote Botanic Research. The saltbush and rhagodia improvement project is supported by Meat and Livestock Australia and Australian Wool Innovation.

## Conflict of interest

The authors declare that the research was conducted in the absence of any commercial or financial relationships that could be construed as a potential conflict of interest.

## Publisher’s note

All claims expressed in this article are solely those of the authors and do not necessarily represent those of their affiliated organizations, or those of the publisher, the editors and the reviewers. Any product that may be evaluated in this article, or claim that may be made by its manufacturer, is not guaranteed or endorsed by the publisher.

## Footnotes

^1^ http://www.R-project.org
